# Contemporary Review of Transcatheter Mitral Valve Interventions for Mitral Regurgitation

**DOI:** 10.3390/life13071511

**Published:** 2023-07-05

**Authors:** Nicholas Chan, Tiffany Dong, Nabil Sabbak, Bo Xu, Tom Kai Ming Wang

**Affiliations:** 1 Section of Cardiovascular Imaging, Department of Cardiovascular Medicine, Heart, Vascular, and Thoracic Institute, Cleveland, OH 44195, USA; 2 Seymour, Paul and Gloria Milstein Division of Cardiology, Department of Medicine, Columbia University Irving Medical Center, New York, NY 10032, USA; 3 Section of Invasive and Interventional Cardiology Section, Department of Cardiovascular Medicine, Heart, Vascular, and Thoracic Institute, Cleveland, OH 44195, USA

**Keywords:** mitral valve, mitral regurgitation, transcatheter mitral interventions, transcatheter therapies, structural heart interventions, echocardiography, computed tomography

## Abstract

Mitral regurgitation (MR) is the most common form of valvular heart disease in the United States, and there are established guidelines for indications for requiring mitral valve surgeries. However, there is an unmet clinical need for a subset of high-risk MR patients, especially those with advanced age, heart failure and/or secondary MR. Following the successes of transcatheter aortic valve replacements, significant advances have occurred over the last decade in transcatheter mitral valve interventions in order to manage these patients in both clinical practice and trials. The three main types of these interventions include a transcatheter edge-to-edge repair, percutaneous mitral annuloplasty (both direct and indirect) and transcatheter mitral valve replacement (including when applied to a prior prosthetic valve, annuloplasty ring and mitral annuloplasty ring). This review aims to discuss the contemporary techniques, evidence, indications, multimodality imaging evaluations and outcomes of the various transcatheter mitral valve interventions.

## 1. Introduction

Mitral regurgitation (MR) is the most common valvular heart disease in the United States, with an estimated prevalence of 2%, further increasing to approximately 9% in those above the age of 75 [1,2], and has traditionally been divided into primary vs. secondary, pending the underlying etiology. In primary or degenerative MR (DMR), the pathology resides in the mitral leaflets. In secondary or function MR (FMR), the pathology usually originates from left atrial or left ventricular (LV) dilation, leading to subsequent leaflet tethering, mitral annular dilation and/or the incomplete coaptation of the mitral valve (MV) [3], portending significant mortality and morbidity in these patients [4]. The natural history of MR is characterized by a latent period prior to the onset of symptoms. MR, if not intervened upon, is associated with poor outcomes, with survival at 5 years presumed to be approximately 50%, previously noted to be undertreated [5,6,7,8]. However, in recent years, due to improved surgical techniques and an increased understanding of the complex MV apparatus anatomy, early intervention has been successful in restoring life expectancy to the level of the general population. When feasible, mitral valve repair and replacement surgeries remain the first-line treatment of severe MR, with recommended DMR indications when there is clinical symptoms, left ventricular systolic dysfunction, and in the absence of these, either a progressive left ventricular dilation and/or dysfunction in at least three studies or at high-volume mitral repair centers, with a >95% expected repair success and surgical mortality of <1% [9]. Mitral valve surgery still comprises the vast majority of mitral procedures, with, for example, 98.6% being recorded in the US National Inpatient Sample Database over the last two decades [10,11]. However, recent advances have led to a rapid surge in options and the uptake of transcatheter mitral valve interventions, especially targeting high-risk or inoperable surgical candidates for both DMR and FMR. Thus, accurate clinical and anatomical assessments, especially amidst advances in multimodality imaging, to risk-stratify and tailor the most optimal therapies for patients are imperative, though challenging. The purpose of this article is to provide a contemporary review of the current transcatheter mitral valve interventions for the treatment of mitral regurgitation, specifically transcatheter edge-to-edge repair, percutaneous annuloplasty and transcatheter mitral valve replacement (Table 1), with the intent of assessing the clinical indications, latest evidence, imaging and outcomes of these procedures.

## 2. Transcatheter Edge-to-Edge Repair

### 2.1. MitraClip for Primary Mitral Regurgitation

Abbott MitraClip was approved by the United States Food and Drug Administration (FDA) in 2013 for the percutaneous reduction in significant (>3+) symptomatic mitral regurgitations due to primary abnormality of the mitral apparatus (degenerative MR) [12]. The main candidates are patients at prohibitive risk for surgery, as determined by a heart team consisting of a cardiac surgeon and cardiologist with expertise in the management of mitral valve (MV) pathology. Compared to surgical candidates, candidates for MitraClip can potentially have more comorbidities that do not preclude them from therapy.

EVEREST (Endovascular Valve Edge-to-Edge Repair Study) II was a multicenter randomized control study consisting of 279 patients with 3–4+ degenerative MR who were randomly assigned in a 2:1 ratio to receive MitraClip vs. surgical repair/replacement. Although MitraClip demonstrated superior safety, as evidenced by the lesser rate of 30-day major adverse events (15% vs. 48%, *p* < 0.001) and improvements in clinical characteristics from baseline (e.g., left ventricular size, New York Heart Association functional class, quality of life measures), the procedure was less effective in reducing MR than conventional surgery at 12 months [13]. Mauri et al. subsequently followed these patients prospectively, whereby at 48 months, there were no differences in mortality (17.4% vs. 17.8%, *p* = 0.914), with a prevalence of 3–4+ MR (21.7% vs. 24.7%, *p* = 0.745). Although patients treated with MitraClip more commonly required surgery to treat residual MR after 1 year (with operative rates for the valvular dysfunction of 20.4% in MitraClip vs. 2.2% in the surgical group, *p* < 0.001), few additional surgeries were required at 4 years (24.8% vs. 5.5%, *p* < 0.001) [14]. The EVEREST II cohort, however, was composed of a very specific patient population of mostly male subjects with a mean age of 67 years and few medical comorbidities, except for heart failure, with a relatively preserved left ventricular ejection fractions amounting to approximately 60%. Thus, Sorajja et al. subsequently examined real-world experience with MitraClip following its approval by the FDA by reviewing the data from the Society of Thoracic Surgeon/American College of Cardiology Transcatheter Valve Therapy (STS/ACC TVT) Registry on patients commercially treated with transcatheter MV repair between 2013 and 2015. In a cohort of 2952 patients, 55% being male and with a mean age of 82 years (significantly older than EVEREST-II patients), with STS risk scores of 6.1% for repair and 9.2% for replacement, the success rate was 91.8%, with an in-hospital mortality of 2.7% [15]. Postprocedurally, there was a significant reduction in MR severity, with 93% having ≤2+ MR and 63.7% having ≤1+ MR. This was profoundly beneficial given that more than moderate MR is a well-established prognostic marker for poor outcomes. The primary pathology was A2-P2 (78%) and 31% had more than one clip, unlike the EVEREST II population. The findings in this sicker, more complex patient population suggested that MitraClip was performed safely and effectively by operators in the United States.

In recent years, a German registry of 800 patients that receiving MitraClip across multiple different sites demonstrated a four-year death rate of 53%. Predictors of mortality postclip included prior aortic valve interventions, renal diseases, New York Heart Association (NYHA) classes III–IV and left ventricular ejection fractions (LVEFs) < 30%, consistent with a sicker patient population [16]. The reason for the profound mortality rate may be that despite the observed benefits in reducing MR and improving the 6 min walk test, there still was a significant disease burden and comorbidities.

### 2.2. MitraClip for Secondary MR

MitraClip may be considered for patients with chronic severe secondary MR related to LV systolic dysfunction (EF < 50%) who have persistent NYHA class II to IV symptoms while on optimal guideline medical therapy (GDMT). Specifically, if the anatomy is suitable on evaluation with TEE, with LVEF between 20 and 50%, end systolic diameter (LVESD) at ≤ 70 mm and pulmonary artery systolic pressure of ≤ 70 mm Hg, it can be a beneficial treatment strategy in a select number of patients (class 2a recommendation) [9].

COAPT and MITRA-FR were the landmark, multicenter randomized control trials that studied the role of MitraClip in functional MR patients. COAPT, which was conducted in North America, consisted of 614 systolic heart failure patients (LVEF 20–50%) with NYHA classes II-IV, symptomatic 3–4+ MR (effective regurgitant orifice (EROA) ≥0.3 cm^2^, regurgitant volume [17] ≥45 cc) on maximally tolerated GDMT ± biventricular pacemaker/defibrillator, who were randomly assigned to receive a transcatheter valve repair plus GDMT vs. GDMT alone. Excluded were those with a LVESD of >70 mm. During the 24-month follow-up, the intervention group experienced a lower rate of heart failure hospitalizations (35.8% vs. 67.9%; hazard ratio (HR) 0.53, 95% confidence interval (CI) 0.40–0.70, *p* < 0.001) and all-cause mortality (29.1% vs. 46.1%; HR 0.62, 95% CI 0.46–0.82, *p* < 0.001), with the rate of freedom from clip-related complications exceeding the prespecified safety threshold [18].

As the European “counterpart” to the COAPT study, MITRA-FR enrolled 304 patients with secondary MR (EROA ≥ 0.2 cm^2^, RV > 30 cc), LVEF between 15 and 40% and symptomatic heart failure, who were not necessarily on optimal GDMT. At the 12-month follow-up, there was no difference in the composite outcome of all-cause mortality, unplanned heart failure hospitalizations (54.6% vs. 51.3%) or its constituent endpoints (24.3% vs. 22.4% for death; 48.7% vs. 47.4% for heart failure hospitalization) [19]. The seemingly conflicting conclusions could be reconciled by the fact that the two studies were composed of patient populations with distinct clinical characteristics. Specifically, MITRA-FR patients had greater chamber dilatation (LV end diastolic volume index of 101 mL/m^2^ vs. 135 mL/m^2^), though a lesser degree of MR (EROA 31 mm^2^ vs. 41 mm^2^) when compared to COAPT. There were significant differences in the rates of procedural complications (14.6% for MITRA-FR vs. 8.5% for COAPT) and proportion of patients with ≥3+ MR post-clip at 12 months (17% for MITRA-FR vs. 5% for COAPT). Per these results, MitraClip appeared to be useful for those with moderate-to-severe MR (EROA ≥ 0.3 cm^2^, RV ≥ 45 cc), with only mild-to-moderate LV dysfunction (LVEF ≥ 20%, LVESD ≤ 70 mm), uncertain for severe LV systolic dysfunction (LVEF < 20%, LVESD > 70 mm) and being futile in those with only mild-to-moderate MR (EROA < 0.3 cm^2^, RV < 45 cc).

### 2.3. Edwards PASCAL System

The Edwards PASCAL transcatheter MV repair system has recently emerged as another viable option for individuals with significant MR but complex, challenging (sub)valvular anatomy. The PASCAL device consists of a central spacer intended to fill the regurgitant orifice area, two broad-contoured paddles designed to reduce stress on leaflets while maximizing leaflet coaptation and two clasps that allow for independent leaflet capture and position fine-tuning, which are especially important in pathologies such as mitral valve prolapse.

Praz et al. described the first-in-man experiences with the PASCAL repair system in a multicenter prospective observational study among a small compassionate-use cohort of 23 patients with moderate to severe MR (26% primary MR, 57% functional MR and 12% mixed) over a 6-month recruitment period, whereby most patients experienced a reduction to grade 2+ or less MR (96%) and improvement in the NYHA functional status (95% class I and II) at 30 days postprocedure [20]. In CLASP, a single-arm prospective study of 190 subjects with symptomatic 3 to 4+ MR (67% functional and 33% degenerative) and appropriate anatomic candidacy who were treated with the PASCAL system, Kaplan–Meier survival was 92%, with 88% experiencing freedom from heart failure hospitalization, a 100% reduction in MR to ≤2+ (82% had 0 to 1+ residual MR) and the predominance of the NYHA class I or II functional status (88%) with a 14-point improvement in the Kansas City cardiomyopathy questionnaire score [21]. Overall complication rates remained low. Recent results from a prospective, multicenter, single-arm registry (PASCAL IID), consisting of 98 symptomatic patients with 3 to 4+ degenerative MR, complex MV anatomy and prohibitive risk for surgery, treated with the PASCAL system, were also promising, in that the implant success rate was high (92.9%), 92.4% had ≤2+ MR (56.1% ≤ 1+ MR) corresponding to symptomatic, functional improvement from baseline and Kaplan–Meier curves for survival, freedom from major adverse events and HF hospitalizations at 6 months were 93.7%, 85.6% and 92.6% [22], respectively. These outcomes suggest that the PASCAL system could even be applied to sicker, frail and elderly inoperable degenerative MR patients with complex anatomies.

### 2.4. PASCAL vs. MitraClip

A few observational studies have compared the outcomes of PASCAL vs. MitraClip for TEER. In a single-center, retrospective, 1:2 propensity-matched cohort of 41 PASCAL and 82 MitraClip cases, Geis et al. noted comparable procedural success, a reduction in MR to ≤2+ and an improvement in the NYHA functional status, as well as similar rates of the composite endpoint of death, HF hospitalization and MV reintervention [23]. The PASCAL cohort did have greater rates of aborted device implantations due to an elevated transmitral gradient (9.8% vs. 1.2%, *p* = 0.04), but, interestingly, had a greater proportion of individuals with none or trace MR at short-term (17.9% vs. 0%, *p* = 0.0081) and 1-year follow-ups (25% vs. 0%, *p* = 0.0016) [23]. In a multicenter, retrospective, 1:1 propensity-matched cohort of 184 patients treated with MitraClip or PASCAL, Schneider et al. found similarly favorable outcomes between the two systems in terms of both safety and performance. The technical success rates did not differ (based on similar proportions of patients with residual MR ≤ 1 or a reduction in MR by at least two grades), and the thirty-day mortality, as well as MR reduction and all-cause mortality at 1-year, were also similar [24].

Most recently, in an interim analysis of the first 180 subjects in the CLASP IID study, a 2:1 randomized control study of individuals with 3 to 4+ degenerative MR at prohibitive surgical risk, demonstrated that the PASCAL system was noninferior to MitraClip for the primary safety and effectiveness endpoints of major adverse event rates (3.4% vs. 4.8%) and MR ≤ 2+ (96.5% vs. 96.8%), respectively [25]. Both groups also experienced significantly improved functional and quality of life outcomes, but more of the PASCAL group had sustained a reduced MR (≤1+) from discharge to the 6-month follow-up (87.2% vs. 83.7%), which was not the case for MitraClip (88.5% vs. 71.2%) [25]. Often during clinical practice, in situations where multiple clips would otherwise be required, the implantation of one PASCAL device (which is larger than MitraClip) often suffices in achieving procedural success. Given the safety profile and efficacy from these findings, which are encouraging for expanding the transcatheter treatment options in prohibitive surgical risk patients with significant symptomatic degenerative MR, the PASCAL device was approved for widespread commercial use in Europe and later in the United States per the FDA for this indication.

### 2.5. Imaging Evaluation

**Figure 1:**
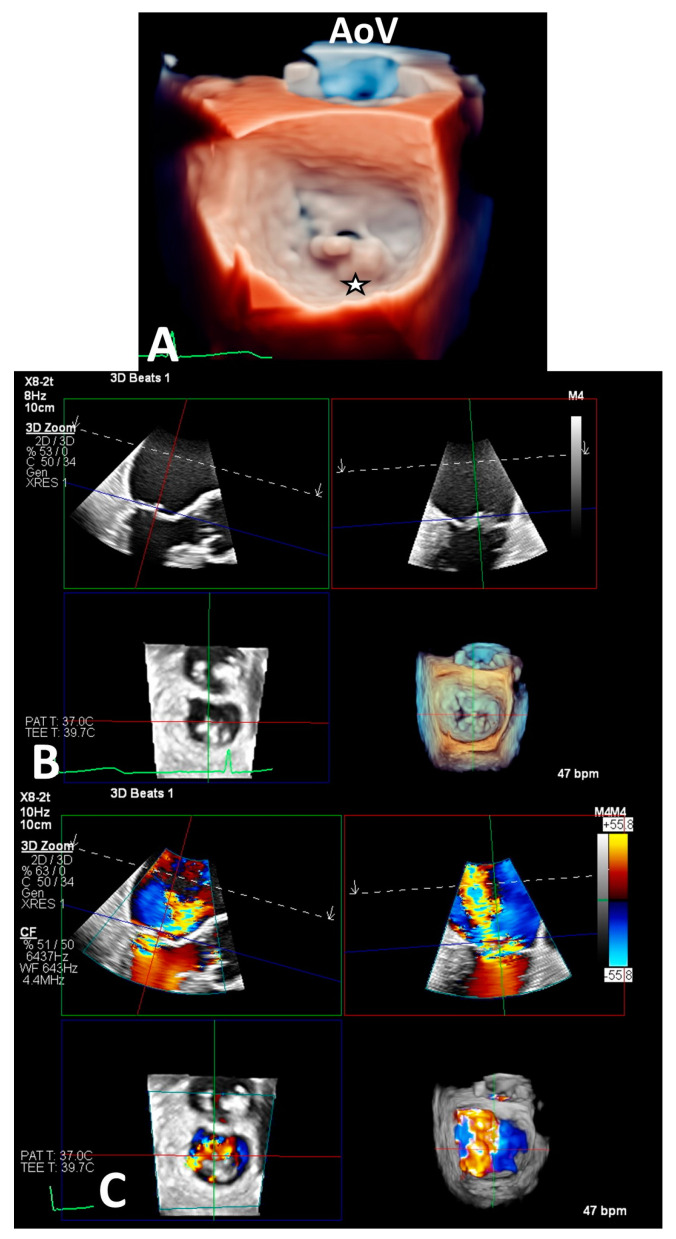
(**A**–**C**) depicts 3D en face imaging of the mitral valve. There is severe primary MR in the context of P2 prolapse and flail (**A**,**B**). The black star in (**A**) shows the site of pathology. AoV denotes the aortic valve for anatomic orientation. (**C**) shows color Doppler interrogation in 3D MPR revealing a significant anterolaterally directed regurgitant jet at the site of pathology.

A thorough imaging evaluation is imperative for the diagnosis and guidance of the device implant, whereby transthoracic and transesophageal echocardiograms are used for the quantification of MR severity on color-flow Doppler and anatomical characterization via both two-dimensional (including biplane) and three-dimensional interrogation and the multiplanar reconstruction (MPR) of the MV (Figure 1). It is important to evaluate the MR mechanism, location, number and severity of MR jets, extent of prolapse, cleft, perforation or calcification in the grasping area, leaflet mobility, length and mitral valve area, which have implications on the procedural outcomes. Selecting patients who are likely to benefit the most was initially based on the criteria set forth in the EVEREST II trial (EVEREST criteria), which included primary MR (ideally at A2-P2), a mitral valve area of ≥ 4 cm^2^, left ventricular ejection fraction of > 25%, end systolic diameter of ≤ 55 mm, flail gap of < 10 mm, flail width of <15 mm, coaptation length of ≥ 2 mm and coaptation depth of < 11 mm. The flail gap, which is the greatest distance between the ventricular side of the flail leaflet segment to the atrial side of the opposing leaflet edge, is measured perpendicular to the plane of the annulus on the four-chamber-long axis and left ventricular outflow tract views on transesophageal echocardiogram (TEE) during systole. The flail width, defined as the width of flail leaflet segment along the coaptation line, is usually measured in the transgastric short axis or commissural views during systole. Evidence of calcification in the grasping area (most distal 7 mm) of anterior and posterior scallops is essential to differentiate from valve fibrosis or thickening. The coaptation length (distance at leaflets) and depth (how deep the valves meet in the LV relative to the mitral valve annulus) are also important, though more relevant for functional MR.

As interventional cardiologists perform more transcatheter edge-to-edge procedures, the suitability criteria for intervention become less complex and stringent. Favorable factors include a single main jet, no calcification at target landing and a mitral valve area of >4 cm^2^, while less-favorable factors including multiple large jets, calcified leaflets, clefts, significant mitral stenosis and intracardiac mass or thrombus [26]. Hence, increasingly more nonclassic patients have been referred, but they remain pertinent, in that any deviations from these criteria suggest that patients may be less likely to respond to or have the best outcomes. When comparing patients who matched and did not match the EVEREST criteria, real life experience showed that despite similar all-cause mortality (28 vs. 27%, *p* = 0.656) during the follow-up of 33 months, the EVEREST patients had significantly lower rates of reintervention (11% vs. 37%, *p* = 0.010) [27]. Postclip, an appreciable proportion (35%) of patients remained with 3+ MR. The lack of long-term sustained benefit in MR reduction in non-EVEREST patients is that they are likely more often of the functional MR phenotype rather than isolated organic or degenerative MR.

### 2.6. Procedure

**Figure 2:**
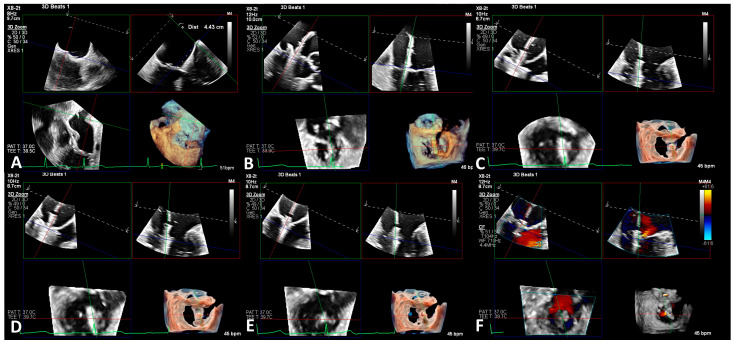
(**A**–**E**) shows the critical steps in mitral transcatheter edge-to-edge repair. A desirable transeptal puncture (TSP) tends to be at the posterior and superior to mid aspect of the fossa ovalis, though this is highly dependent on interatrial septal anatomy, the SVC/IVC trajectory and the relationship of the fossa ovalis to the mitral valve. The TSP shown in (**A**) is at the mid/superior portion of the fossa ovalis. A height of >4.0 cm above the medial commissure of the mitral valve is typically necessary. (**B**) shows the clip trajectory, as well as orientation prior to bringing the clip to the ventricular side of the mitral valve. (**C**) shows clip orientation as well as the grasping view in the green box (upper left, within (**C**)). In (**D**), the anterior and posterior mitral leaflets were captured with the clip arms, and the clip was subsequently closed (**E**). (**F**) is a 3D color Doppler interrogation of the mitral valve after deployment of one clip with trace residual MR.

Both MitraClip and PASCAL devices have similar procedural techniques, and periprocedural imaging plays an important role. Multimodality imaging via TEE with 3D MPR and preferably biplane fluoroscopy are imperative for a successful TEER. The cardiac imager and structural interventionalist should establish a language that effectively communicates the relationship between the clip and intended grasping plane. This language should acknowledge lateral vs. medial and anterior vs. posterior maneuvers. The “grasping view” should be displayed during device positioning, whereby the biplane is applied through a commissural view on TEE, generating a short axis and 3D MPR all in one screen. The structural cardiologist should make adjustments upon the intended axis and appreciate this on the echocardiographic images. Biplane fluoroscopy may further inform movements that are anteriorly vs. posteriorly appreciated in the RAO, and septally vs. laterally appreciated in the LAO. Fluoroscopy is particularly helpful in further visualizing the movement of device components during leaflet grasping and release. Figure 2 depicts the key steps of this procedure with TEE and live 3D MPR guidance.

The trans-septal puncture is an important step that should be performed safely under TEE guidance. Imaging should verify a posterior puncture in the short-axis view so as to avoid proximity to the aortic valve, and superior in the bicaval view for approach with the device delivery system with an optimal height of at least 4.0 cm above the mitral annulus. A BRK needle through an SL-braided trans-septal guiding introducer (St. Jude Medical, Fullerton, CA, USA) may be utilized for this step, although there may be slight variations in the equipment utilized. Once trans-septal access is obtained, a stiff wire such as an Amplatz may be advanced into the left upper pulmonary vein. This provides adequate purchase and stability to advance the steerable guide catheter with a dilator into the left atrium (LA). Once the wire and dilator are removed, the clip deliver system may be advanced into the LA, with careful TEE and fluoroscopic visualization in order to avoid injury to the LA.

The grasping view with 3D MPR as referenced above is maintained as the clip is maneuvered medial to lateral and anterior to posterior. The clip must be positioned above the mitral annulus perpendicular to the coaptation of the leaflets upon the jet origin identified through the proximal isovelocity surface area (PISA). Again, communication between the cardiac imager and structural cardiologist is key. This requires a mutual understanding of 3D anatomical relationships and the coregistration of fluoroscopic and echocardiographic movements of the device. Once properly positioned, the arms of the clip may be opened, ensuring perpendicular orientation to the leaflet coaptation. The clip may be advanced into the left ventricle with the arms open or closed, depending on operator preference. It may be necessary for the readjustment of the clip orientation if advanced with arms open into the left ventricle [28]. Breath-holding on the ventilator may be utilized to minimize motion during final adjustments. It is crucial to ensure optimal positioning before grasping, whereby the clip arms are perpendicular to the line of the coaptation upon the PISA, with the anterior and posterior leaflets visualized freely mobile above the clip arms. At this point, the grippers may be deployed, capturing the leaflets between the grippers and arms. Leaflet capture may be confirmed by partially closing the clip. A thorough echocardiographic evaluation is necessary before device release to ensure proper stability and result. Residual MR should be assessed to ensure the intended PISA was captured. If there is a long segment of MR along the coaptation line of coaptation, multiple jets or a complex MR mechanism, there is a higher risk of significant residual MR not adequately treated by the clip, as well as mitral stenosis, especially when multiple clips are utilized. Left atrial waveforms with particular attention paid to V-waves may further inform such decision making. Additional clips may increase the mitral valve gradient; hence, a Doppler measurement should confirm a gradient of < 5 mmHg, or else patients may suffer from hemodynamic consequences of mitral stenosis.

## 3. Percutaneous Mitral Annuloplasty

There are two main categories of percutaneous mitral annuloplasty: direct and indirect. Direct annuloplasty is characterized by the attachment of the ring/band to mitral annulus, thus, closely resembling surgical annuloplasty. However, the procedure is more complicated and imaging-intensive due to the need for the precise placement of anchors on a moving heart [29]. Indirect annuloplasty works by modifying the shape of an adjacent structure, such as the coronary sinus, to improve coaptation. Though less technically complicated, the mitral annulus and coronary sinus are not necessarily coplanar; thus, movements in the coronary sinus do not necessarily correspond to changes in the shape of the mitral annulus in a 1:1 fashion. Both procedures are performed under fluoroscopic guidance, with echocardiography being mandatory to evaluate the severity and mechanism of MR during the pre- and postoperative interrogations.

### 3.1. Direct Annuloplasty

The most widely implanted direct annuloplasty device for secondary MR is the Cardioband, a flexible polyester sleeve through which 12–17 sequential anchors at 8 mm increments are implanted on the posterior annulus from the anterolateral to posteromedial commissure [30]. The entire system is tensioned to the spool to reduce the mitral annular diameter and circumference to eliminate significant mitral regurgitation. In a single-arm prospective multicenter trial experience of 60 high-surgical-risk patients with moderate to severe secondary MR treated with the Cardioband system, Messika-Zeitoun et al. noted, through a Kaplan–Meier analysis a 1-year overall survival, survival free of heart failure readmission and the survival of reintervention rates of 87%, 66% and 78%, respectively, as well as a reduction in MR severity to moderate or less and an improvement in functional status in the majority of patients [30].

Another device under development is the Millipede, a complete semirigid ring with eight anchors that is of adjustable size, fully repositionable and retrievable up until final deployment and embedded with an intracardiac echocardiography catheter as part of the delivery system that enables the live visualization of anchor placement by the operator [31]. Early experience in a series of seven patients showed an average reduction in septal lateral dimension by 31% and a decrease of 3–4+ MR at baseline to 0 to 1+ at discharge and 30 days later [31]. Finally, the AMEND system, which can reform through the catheter into a D-shaped semirigid complete ring with extruding anchors and is delivered via a transapical approach, has also been performed in humans [17], but further research is needed.

### 3.2. Indirect Annuloplasty

**Figure 3:**
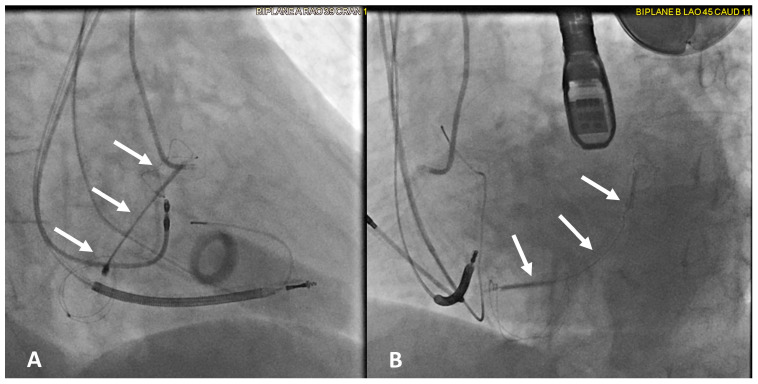
(**A**,**B**): Carillon^®^ Device RAO (**A**) and LAO (**B**) fluoroscopic views of the Carillon^®^ Mitral Contour System™ device implanted in the coronary sinus. The arrows delineate the position of the Carillon device^®^.

The Carillon system is the most commonly utilized transcatheter indirect annuloplasty technique, consisting of two self-expanding anchors (at the proximal coronary sinus and great cardiac vein) connected with a fixed-length nitinol cable, which is inserted via a puncture in the jugular vein and a delivery catheter under fluoroscopic guidance (Figure 3). This device was studied in three prospective studies: the TITAN, TITAN II and REDUCE-FMR studies [32]. In the initial TITAN study, a prospective, nonrandomized, nonblinded multicenter trial, 53 patients with a symptomatic, dilated ischemic or nonischemic cardiomyopathy of LVEF < 40% on guideline-directed medical therapy and at least 2+ functional MR were included, 17 of whom did not receive the Carillon device due to anatomic considerations. It was shown that the implanted cohort had significant reductions in regurgitation volume (34.5 mL/beat to 17.4 mL/beat *p* < 0.001) as well as LV diastolic (208.5 mL to 178.9 mL, *p* = 0.015) and systolic volumes (151.8 to 120.7 mL, *p* = 0.015) at 12 months, compared with progressive LV dilation in the nonimplant group, and markedly improved 6 min walk tests at 24 months [33]. The subsequent TITAN II trial, which followed up only on 36 patients who received the Carillon device, confirmed the safety profile of the device and its efficacy in improving the functional class and 6 min walk tests [34]. Most recently, the REDUCE-FMR trial, a randomized double-blind, proof-of-concept study of the Carillon device, further confirmed the device’s benefits in improving the MR volume (−7.1 mL/beat vs. +3.3 mL/beat) and LV end-diastolic (−10.4 mL vs. +6.5 mL) and systolic volumes (−6.2 mL vs. +6.1 mL) when compared to the control sham group, with corresponding improvements in the NYHA functional status [35].

## 4. Transcatheter Mitral Valve Replacement

**Figure 4:**
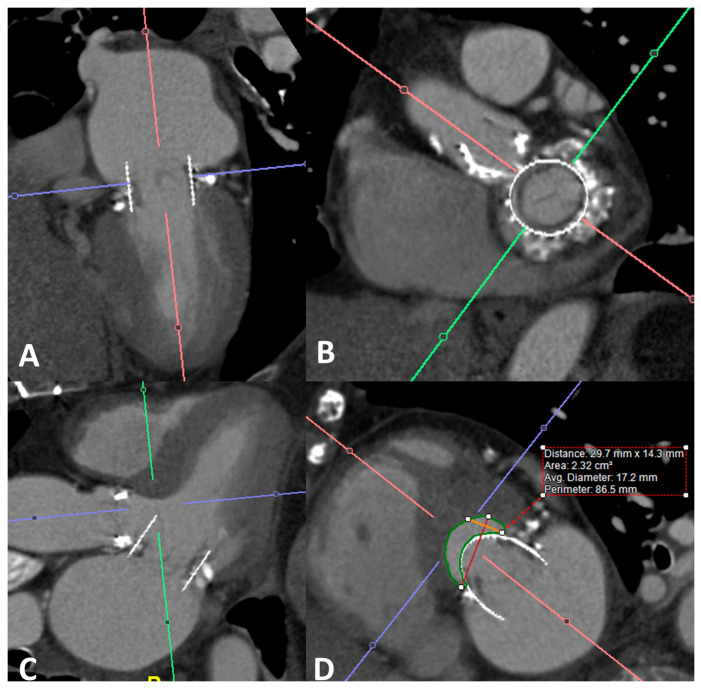
(**A**–**D**) depict multiplanar reconstruction (MPR) of a 4D CT scan (no dose modulation) performed for valve-in-valve transcatheter mitral valve replacement (ViV TMVR) procedural planning. The patient had severe MR due to bioprosthetic mitral valve degeneration. Images were reconstructed in an early systolic phase with dedicated software to simulate implantation of a 29 mm bioprosthesis off-set to match the length of the anterior mitral valve leaflet. (**A**,**B**) show the simulated bioprosthesis in the CT equivalent of 2-chamber and short-axis views, respectively. (**C**,**D**) were reconstructed to measure the neo-LVOT, measured at 2.3 cm^2^, in this example, which was deemed suitable for ViV-TMVR without the need for leaflet modification.

**Figure 5:**
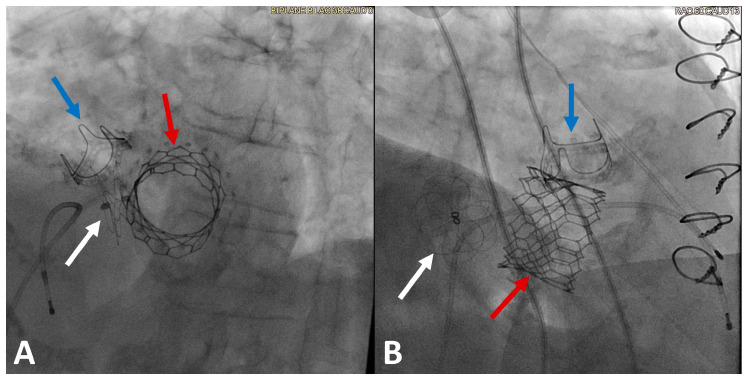
Shows fluoroscopic images LAO (**A**) and RAO (**B**) of ViV TMVR with a 29 Sapien S3 prosthesis. In this case, the iatrogenic ASD was closed due to intermittent right-to-left shunting that was seen on intraprocedural intracardiac ultrasound. The red arrows mark the ViV TMVR, the blue arrows mark the aortic bioprosthesis and the white arrows mark the atrial septal occluder device.

**Figure 6:**
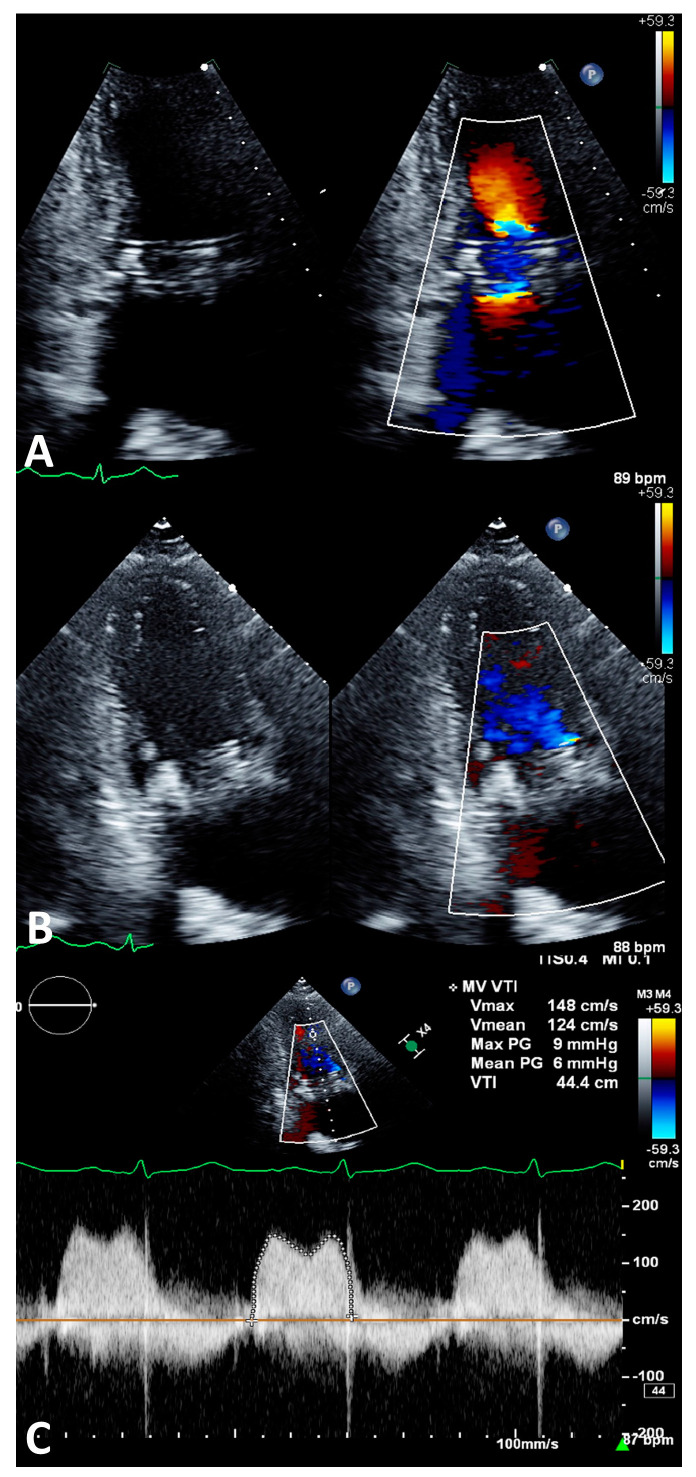
(**A**–**C**) shows postprocedural transthoracic echocardiographic images following ViV TMVR. (**A**) shows a 2-chamber color-compare image in early diastole with mild flow acceleration across the transcatheter mitral valve. (**B**) shows a 3-chamber color-compare image in early systole without significant MR or LVOT flow acceleration appreciated. (**C**) shows a mean transmitral gradient of 6 mmHg at a heart rate of 87 beats per minute.

The transcatheter mitral valve replacement (TMVR) is performed through the implantation of either an aortic or mitral transcatheter heart valve (THV) in the mitral position. Although not specifically designed for the mitral position, the aortic THV has been successfully employed in patients with failed bioprostheses (mitral valve-in-valve (MViV)), failed prosthetic rings and bands (mitral valve-in-ring (MViR)) and native calcified valves (valve in mitral annular calcification (ViMAC)). On the other hand, newer TMVR devices with mitral THVs have primarily been used for native noncalcified mitral valves. A preoperative evaluation is critical through the utilization of both transesophageal echocardiographs to evaluate the severity and mechanism for prosthetic mitral stenosis and/or regurgitation along with four-dimensional retrospectively gated cardiac computed tomography to evaluate for prosthesis sizing and the possibility of left ventricular outflow tract obstruction using multiplanar reconstruction (Figure 4). Both fluoroscopy (Figure 5) and transthoracic echocardiography (Figure 6) are important in the deployment of the new valve and the postdeployment evaluation of valvular function, respectively.

### 4.1. Valve in Valve

Based on data from VIVID and TVT registries, where MViV was performed via a trans-septal approach in 18.5% and 49% of patients, respectively, the 30-day mortality was approximately 8–9%. Later, the MITRAL trial, which relied exclusively on a trans-septal technique, was able to further decrease the 30-day mortality to 3.3% [36]. However, this cohort was a highly selected population of patients with extremely experienced operators. To determine whether this lower mortality could be reproduced in the real world, especially on longer follow-up times, Whisenant et al. evaluated more contemporary outcomes with the Sapien 3 valve from the TVT registry. In 1529 patients hospitalized between 2015 and 2019 for MViV (1326 trans-septal and 203 transapical), procedural success was high (96.8%), with an all-cause mortality of 5.4% at 30 days and 16.7% at 1 year [37]. Though no differences were observed in the technical success rate of either approach, the trans-septal approach was associated with a lower mortality at 1 year (15.8% vs. 21.7%) with a hazard ratio of 0.67, thus, highlighting the importance of choosing the trans-septal approach in patients with a favorable anatomy [37]. MViV also led to early, sustained and clinically meaningful improvements in heart failure symptoms, as well as valve performance. In comparison, for the MITRAL trial, the mortality at 1 year was 3.3% (unchanged from 30-day outcomes) and 6.7% at 2 and 3 years [36]. Based on the available data, MViV was, thus, FDA approved for high-risk patients in June 2017 [38], but is actively being investigated in the ongoing PARTNER 3 trial for intermediate-surgical-risk patients, which consists of a prospective registry of 50 patients at 16 sites in the United States and Canada that recently completed enrollment in 2021 [39].

From an imaging standpoint, it is important to note that in 60–80% of patients treated successfully with MViV for a failed surgical bioprosthesis, abnormally increased residual transvalvular gradients were measured in transthoracic echocardiography before discharge or at 30 days, despite being within normal limits in a routine periprocedural transoesophageal echo. A few in vitro studies reported a substantial impact in actual MViV frame geometry (such as eccentricity, a nonround shape and underexpansion) on the altered transvalvular flow characteristics, and a large field-of-view intravascular ultrasound (IVUS) offers a unique tomographic perspective for a direct periprocedural measure of the MViV stent frame and leaflet geometry [40,41]. It is the actual three-dimensional expansion of the MViV stent frame that determines the pattern of the restored blood flow and the long-term outcomes of valve-in-valve deployment, and actual MViV expansion may differ significantly from the nominal, and there is no valid periprocedural measure to date. The periprocedural use of a large field-of-view IVUS may offer accurate and online measurements of the actual expansion of MViV deployed [40,41].

### 4.2. Valve in Ring

Based on the VIVID and TVT registries, early experiences with MViR patients had higher 30-day mortality of approximately 11–13%. In the MITRAL trial, which switched from a transapical to exclusively trans-septal approach, the 30-day mortality was significantly reduced to 6.7% [42]. The 1-year mortality of 23.3% was lower than predicted by the STS score of 7.6% [42] and similar to that of patients who were treated with MitraClip in the US based on the TVT registry [15], which was a favorable result, given the very well-established track record of transcatheter edge-to-edge repair. At 2 years, the mortality rate was 43.3%, which was similar to devices designed for the mitral valve, such as Tendyne, discussed in a later section. Patients receiving smaller-sized THVs with rigid rings were noted to have higher gradients. A subsequent analysis of contemporary data from a TVT database with Sapien 3 MViR found that the 30-day all-cause mortality decreased dramatically over time from approximately 11% in 2015–2016 to 6% in 2020, reproducing better outcomes with mortality rates similar to the MITRAL trial. The access route was also switched from being mainly transapical to transeptal. Based on these data, the FDA approved MViR for high-risk patients in May 2021 [38].

### 4.3. Valve in MAC

Patients with MAC represent the highest-risk population, even prior to their presentation with valvular dysfunction. Multimodality imaging incorporating echocardiography and cardiac computed tomography is being increasingly recognized as being key for the assessment and grading of MAC and associated valve dysfunction [43]. To improve the MAC evaluation, a novel computed-tomography-based MAC score has been shown to predict outcomes among 334 patients with mitral valve dysfunction undergoing mitral valve surgery [44]. From the Framingham Heart Study, which consisted of 1197 patients who had a transthoracic echo with an average follow-up of 16 years, these individuals were often older females with significant cardiovascular comorbidities, such as hypertension, atrial fibrillation, stroke, coronary and peripheral artery disease, that elevated their risk for mortality [45]. Coupled with the technical challenges during surgery and potential complications from calcium deposition (such as posterior LV wall rupture), these risk factors increase the mortality to >20%. Thus, this cohort of patients are often left untreated and have traditionally been understudied until recently. A second study found that among 24,414 patients who underwent a clinically indicated echo, MAC was present in 23% (*n* = 5502) of the population [46]. Patients with MAC and MV dysfunction had a higher prevalence of mitral stenosis (MS) compared to those with MV dysfunction without MAC (6.6% vs. <1%). From a mortality standpoint, isolated MAC without MV dysfunction already had compromised outcomes (similar to those with MV dysfunction without MAC) compared to those without MAC or mitral valve dysfunction; 1-year Kaplan–Meier survival was 86–87% vs. 92%, with an HR of 1.40 (95% CI 1.31–1.49, *p* < 0.001) [46]. However, when MAC was associated with MV dysfunction, the 1-year survival was even poorer at 76%, with an HR of 1.79 (95% CI 1.58–2.01, *p* < 0.001) [46]. From a further analysis of this same cohort, during a median follow-up of 3.2 years, patients with MAC and severe mitral valve dysfunction who received intervention had higher survival than those without intervention (90% vs. 72% at 1 year; 55% vs. 35% at 4 year). The MV intervention was, thus, found to be an independent predictor of lower mortality, with a hazard ratio of 0.66 [47]. Another study also found mitral valve interventions to be associated with improved prognosis after propensity score matching, and the key predictors of mortality being a higher Charlson comorbidity index and frailty [48].

However, outcomes from the early experience with ViMAC were not ideal. From the TMVR in a MAC global registry, which consisted of 116 MAC patients with high STS scores of 15.3%, the 30-day and 1-year mortalities were 25% and 53.7%, respectively [49]. Similarly, from the multicenter TMVR registry led by the Cedars Sinai team, which consisted of 58 MAC patients, the 30-day mortality was even higher at 34.5% [50]. This could be attributed to three main challenges/complications with ViMAC: paravalvular leak with associated hemolysis, valve embolization and LVOT obstruction, the latter two of which can be predicted and, thus, prevented with improved temporal outcomes. LVOT obstruction was readily established as the strongest independent predictor of 1-year mortality for TMVR with an adjusted hazard ratio of 2.63 [49]. The two main ways to prevent LVOT obstruction are septal reduction strategies and anterior leaflet strategies. Septal reduction strategies include alcohol septal ablation, a concept generated in the MITRAL trial [51], and radiofrequency septal ablation [52]. Anterior leaflet strategies include surgical resection (MITRAL and SITRAL trials) and percutaneous laceration (LAMPOON trial). From a multicenter registry experience of preemptive alcohol ablation to prevent LVOT obstruction, there was an incremental increase in the size of the LVOT space, where the baseline median Neo-LVOT was 85.1 mm^2^, with a median increase of 111.2 mm^2^ [52]. In cases where ViMAC is high risk, if the Neo-LVOT < 200 m^2^, the optimal strategy to prevent LVOT obstruction is determined by measuring the Skirt Neo-LVOT, whereby the septal reduction strategy is considered if <200 mm^2^ vs. LAMPOON-facilitated transeptal ViMAC if >200 mm^2^ [53]. In the MITRAL trial using alcohol septal ablation, there was an improvement in patient outcomes, whereby in a population with an STS score of 8.6%, trans-septal ViMAC had a 30-day mortality of 6.7%, compared to 21.4% for transatrial access. The 1-year mortality of 33% was not dissimilar to the MitraClip TVT registry, especially for secondary MR (31.2%) [15,54], while the 2-year mortality of 39.9% was similar to the transcatheter aortic valve (TAVR) experience from the PARTNER 1 trial (43.3%) [55]; the 3-year survival was 50% in a high-risk population. Furthermore, the mean mitral valve gradient remained stable at 2 years. Given these encouraging trials, the MITRAL II Pivotal trial is being conducted for high-risk surgical patients with severe MAC MS (MV area ≤ 1.5 cm^2^) or ≥3+ MR with NYHA class II or greater symptoms. The two arms are ViMAC with 100% trans-septal access + preemptive LVOT obstruction, for those with favorable anatomy and a CT MAC score ≥ 7 (*n* = 110), vs. natural history (*n* = 100) for noncandidates for intervention. The primary endpoint of interest will be all-cause death and heart failure hospitalization at 1 year.

### 4.4. Differences in Outcomes of Valve in Valve vs. Valve in Ring vs. Valve in MAC

Early experience with TMVR in the United States using aortic THV, which started with the off-label use of aortic THV, was first highlighted by the TVT registry from 2013 to 2017. There was a significant difference in outcomes of patients cleared for MViV, who had the lowest in-hospital and 30-day mortality when compared with MViR and ViMAC [56]. Although various factors, including distinct patient populations and procedural techniques, could have explained the varied outcomes between these three groups, such differences persisted in multiple subsequent studies. In a single-center study of 91 patients in France, Urena et al. observed that ViMAC had the highest mortality at 1 year (41.7%) and 2 years (58.4%) when compared to MViV (13.2% at 1 year; 29.4% at 2 years) and MViR (12.7% at 1 year; 24.5% at 2 years) [57]. Based on Cedar Sinai’s multicenter TMVR registry of 521 patients, ViMAC also had the highest 1-year mortality (62.8%) compared to MViR (30.6%) and MViV (14.0%) individuals [50]. The MITRAL trial confirmed these trends, whereby the three-year survival was significantly better for MViV (93.3%) than MViR and MViV (<60%) patients.

### 4.5. Valve in Native Noncalcified Valve

There has been extensive ongoing research into the use of dedicated THVs for native noncalcified MV diseases. The most well-studied and only mitral THV approved in Europe is the Tendyne, which is a transapically deployed trileaflet porcine bioprosthetic valve with its outer frame contoured to mitral annulus and available in multiple sizes and profiles to address a broad range of patient anatomies. From an initial report of the first 100 patients from the Tendyne Global Feasibility Study, 90% of whom had secondary MR, with a mean STS score of 7.9% and EF of 46.6%, the intraprocedural mortality was 0% and 30-day mortality was 6%, which was the first time that a transcatheter mitral device resulted in a 30-day mortality lower than predicted by the STS score [58]. At 12 months, there was an impressive reduction in the degree of MR with 98.4% of individuals having none too trivial MR at 12 months and sustained significant improvement in symptoms [58]. The 1-year mortality of 26% was similar to MitraClip per the TVT registry (24.7% for degenerative MR and 31% for functional MR) and TAVR per the PARTNER 1A trial (24%) [58]. Due to the favorable outcomes of this device, it received approval on 30/1/2020 [59]. Furthermore, the 2-year mortality was 41.6%, with a continued sustained improvement in MR, clinical symptoms and quality of life, as well as a significant reduction in the heart failure hospitalization rate (0.51 events per patient-year postoperation vs. 1.30 events per patient-year pre-operation) [60].

Early experience with Tendyne in MAC also demonstrated promising results. Per Sarajja et al., the first nine patients who received Tendyne in MAC who had an STS score of 7.4%, had a 30-day mortality of 0% [61]. A subsequent analysis of the first 20 patients showed a 1-year all-cause mortality of 40%, which was almost identical to the 1-year mortality of 39.9% from the original MITRAL trial, despite a higher-risk patient population [62]. Currently, SUMMIT (Safety and Effectiveness of Using the Tendyne Mitral Valve System for the Treatment of Symptomatic Mitral Regurgitation) is an ongoing study recruiting patients with symptomatic grade III/IV MR or severe MAC, deemed by the heart team to be more suitable for the transcatheter treatment than surgery [63]. For those individuals with MV anatomy and indications appropriate for transcatheter repair, they were randomized 1:1 to receive Tendyne or MitraClip; otherwise, subjects were directly offered Tendyne.

## 5. Mitral Paravalvular Leak Closure

Paravalvular leaks (PVL) occur in approximately 5–17% of surgical valves deployed in the mitral valve position, and most (74%) are diagnosed in the first year postoperatively [64,65,66,67,68,69]. The most common risk factors associated with mitral PVL include a prior history of endocarditis, significant mitral annular calcification, corticosteroid use, mechanical prosthesis (as opposed to biological), supra-annular valves and the type of surgical techniques used (continuous > interrupted sutures) [70,71,72,73,74]. Mitral PVLs can be difficult to diagnose, given that they are only symptomatic in approximately 1–5% of patients, but individuals can present with signs/symptoms and sequelae of heart failure, hemolytic anemia (due to blood shear stress) and infective endocarditis, which are all indications for percutaneous closure if anatomy is favorable. Contraindications include active infective endocarditis and significant dehiscence involving more than 1/3 to ¼ of the valve ring.

**Figure 7:**
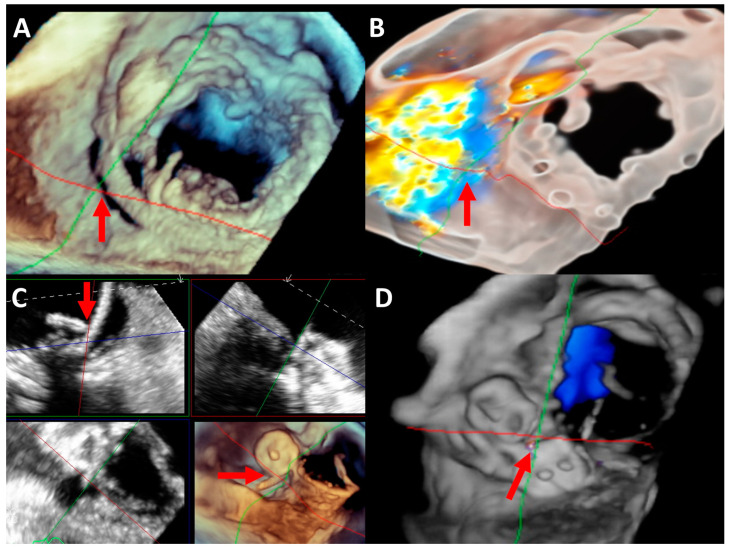
Three-dimensional transesophageal echocardiography with evaluation and guidance of paravalvular leak closure. (**A**,**B**) show preprocedural evaluation with and without color Doppler, identifying severe paravalvular leak on the posterolateral aspect of the bioprosthetic mitral valve (arrows). (**C**) shows during procedure after one vascular plug is deployed, the wire crossing the defect below the prior plug in anticipation for the second plug to be deployed localized with live multiplanar reconstruction imaging (arrow). (**D**) shows the final result after 5 vascular plugs were deployed for the paravalvular leak with color Doppler showing trivial residual regurgitation (arrow).

Multimodality imaging plays an important role in both diagnosis and management. Transthoracic echocardiography, which can assess transvalvular flow/hemodynamics and lesions, is the primary modality for both the diagnosis (though clues can be subtle due to limitations from significant acoustic shadowing) and postprocedural follow-up. Transesophageal echocardiography, with the use of 3D imaging, is the gold standard for determining the location and severity of defects (based on color flow area, jet density and contour, pulmonary arterial systolic pressure and venous flow, vena contracta, the circumferential extent of PVL, MR volume/fraction and EROA), and is the critical modality for the preprocedural evaluation, intraprocedural guidance and immediate postprocedural evaluation (Figure 7) [64]. Cardiac computed tomography (CT) cannot only be used for anatomical characterization as part of preprocedural planning, but also be coregistered with fluoroscopy (CT–fluoroscopy fusion imaging) during the procedure to assist the interventionist with facilitating access, wire crossing and device deployment [75,76]. Cardiac magnetic resonance allows for an accurate volumetric assessment and the quantification of multiple defects [64].

Under imaging guidance, mitral PVL closure is performed via one of three methods, of which the choice of approach can be influenced by the localization and multiplicity of PVL. In the most common anterograde approach, after securing femoral venous access, a trans-septal puncture is performed to advance the wire to the LA, then LV and further into the ascending aorta, and a venoarterial loop is established by capturing the wire using a snare in the ascending aorta (most commonly at the aortic arch entrance) introduced from the femoral artery. The delivery sheath is then advanced over the venoarterial loop from the venous side to the LV through the leak, holding both ends of the wire tight with the help of two mosquito clamps, and once in place, the closure device is transported and deployed. In the retrograde approach, after securing the femoral arterial access, the wire is advanced through the aorta into the LV and then LA, and an arteriovenous loop is established by capturing the wire in the LA using a snare advanced from the femoral vein into the LA with a trans-septal puncture. The delivery sheath is then advanced over the venoarterial loop to the LA and to the LV through the leak and the closure device is deployed. It is important to achieve a good alignment of the delivery sheath with the defect in an orthogonal axis to avoid the incomplete closure of the PVL or interference with the mitral prosthesis. The third, and least common, is the transapical approach, which offers less wiring difficulty and lower resistance across the PVL, but at the cost of higher complication rates than the anterograde or retrograde approach [77]. Regardless of approach, after occluder deployment, it is important to check for device positioning in relation to prosthesis on fluoroscopy, the degree of residual MR on color Doppler, transvalvular gradients to rule out any iatrogenic prosthetic valve obstructions and the presence of new pericardial effusions.

Despite the technical challenges of the procedure and global differences in the availability of occluder devices, outcomes for transcatheter mitral PVL closure have shown promise. In a large multicenter Spanish registry of 469 patients who underwent PVL closure with different types of devices (including the Amplatzer AVP III), of which approximately 70% involved the mitral prosthesis, the technical and procedural success rates were reported as 86.6% and 73.2%, respectively, with low rates of major complications [78]. In another multicenter registry from the United Kingdom and Ireland consisting of 259 patients undergoing percutaneous PVL closure, of which 44% were MV only, successful device implantation occurred in 91% of patients [79]. Hospital mortality was 2.9% for elective procedures and 6.8% for urgent in-hospital cases [79]. Postintervention, PVL was mild to nonexistent in 74.7% of individuals, and the NYHA class significantly improved at follow-up [79]. Most recently, a multicenter, multination study in Europe of 136 patients who underwent percutaneous PVL closure (67.6% mitral) demonstrated a high success rate associated with clinical improvement, with an all-cause mortality of 7.4%, declines in the proportion of patients with NYHA classes III/IV or requiring hemolysis-related blood transfusion, declined significantly, and resulted in relatively low rates of serious complications [80]. More research regarding potential benefits of various device types is needed and currently in progress.

## 6. Conclusions

Transcatheter mitral interventions have grown in number and advanced over the last decade, becoming an integral part of structural heart interventions, targeting MR patients with high or prohibitive surgical risk. Transcatheter edge-to-edge repair is the most widely performed, while other techniques, including annuloplasty, TMVR and paravalvular leak closure, have critical roles in their specific patient groups of FMR, prosthetic valvular regurgitation or stenosis and paravalvular MR, respectively. We now have over 10 years for DMR and over 5 years for FMR worth of results for transcatheter mitral valve interventions, especially for edge-to-edge, and high rates of improving procedural feasibility (95+%) and efficacy to reduce MR (none to moderate over 80%) reported in contemporary trials and registries. There is increasing evidence supporting the clinical roles for these techniques, and also great emphasis on the utility of echocardiography and multimodality imaging in the evaluation and intraprocedural guidance for these procedures. A multidisciplinary heart team remains central to both the decision making to undertake and actually perform the transcatheter mitral intervention in order to improve the clinical outcomes of these often challenging cohorts of MR patients.

## Figures and Tables

**Table 1:** Summary of transcatheter techniques to treat mitral regurgitation.

Technique	Transcatheter Edge-to-Edge Repair	Direct Annuloplasty	Indirect Annuloplasty	Transcatheter Mitral Valve Replacement ^a^
**Device(s)**	MitraClip (Abbot) Pascal (Edwards)	Cardioband (Edwards)	Carillon (Cardiac Dimensions)	AltaValve (4C Medical Technologies) Caisson (Caisson Interventional) Evoque (Edwards) HighLife (HighLife Medical Inc.) Intrepid (Medtronic) NAVI (NaviGate) Sapien 3 (Edwards) Tendyne (Abbott) Tiara (Neovasc)
**Mechanism**	Apposition of leaflets to reduce malcoaptation gap	Anchors are placed along posterior MV annulus and the band is then “cinched”	Cinching with Nitinol wire and anchors from the coronary sinus	Deploying a prosthetic valve within a prior MVR, MVr or MAC
**Indications**	High-risk or prohibitive-risk surgical candidates with primary > 3+ MR or secondary > 3+ MR	Secondary MR	Secondary MR	Primary (including MVR, MVr, MAC) and secondary MR
**Cautions**	- Multiple MR jets - MVA < 4 cm^2^ or significant MS - Calcified leaflets in grasping zone - Thrombosis	- Significant MAC - Significant asymmetric stenting leading to restriction of posterior leaflet	- Existing CS lead/device - Prior MVR or MV ring	- Selective to the patient’s anatomy and respective device - More optimal results in prior MVR > MV ring > MAC

^a^ Some TMVR devices are still under investigation and/or available on off-label use. Abbreviations: CS—coronary sinus; MAC—mitral annular calcification; MR—mitral regurgitation; MV—mitral valve; MVr—mitral valve repair; MVR—mitral valve replacement.

## Data Availability

Not applicable.

## References

[1] Asgar A.W., Mack M.J., Stone G.W. (2015). Secondary mitral regurgitation in heart failure: Pathophysiology, prognosis, and therapeutic considerations. J. Am. Coll. Cardiol..

[2] Nkomo V.T., Gardin J.M., Skelton T.N., Gottdiener J.S., Scott C.G., Enriquez-Sarano M. (2006). Burden of valvular heart diseases: A population-based study. Lancet.

[3] de Marchena E., Badiye A., Robalino G., Junttila J., Atapattu S., Nakamura M., De Canniere D., Salerno T. (2011). Respective Prevalence of the Different Carpentier Classes of Mitral Regurgitation: A Stepping Stone for Future Therapeutic Research and Development. J. Card. Surg..

[4] Rossi A., Dini F.L., Faggiano P., Agricola E., Cicoira M., Frattini S., Simioniuc A., Gullace M., Ghio S., Enriquez-Sarano M. (2011). Independent prognostic value of functional mitral regurgitation in patients with heart failure. A quantitative analysis of 1256 patients with ischaemic and non-ischaemic dilated cardiomyopathy. Heart.

[5] Delahaye J.P., Gare J.P., Viguier E., Delahaye F., De Gevigney G., Milon H. (1991). Natural history of severe mitral regurgitation. Eur. Heart J..

[6] Enriquez-Sarano M., Avierinos J.-F., Messika-Zeitoun D., Detaint D., Capps M., Nkomo V., Scott C., Schaff H.V., Tajik A.J. (2005). Quantitative Determinants of the Outcome of Asymptomatic Mitral Regurgitation. N. Engl. J. Med..

[7] Enriquez-Sarano M., Sundt T.M. (2010). Early surgery is recommended for mitral regurgitation. Circulation.

[8] Head S.J., van Leeuwen W.J., Van Mieghem N.M., Kappetein A.P. (2014). Surgical or transcatheter mitral valve intervention: Complex disease requires complex decisions. Eurointervention J. EuroPCR Collab. Work. Group Interv. Cardiol. Eur. Soc. Cardiol..

[9] Otto C.M., Nishimura R.A., Bonow R.O., Carabello B.A., Erwin J.P., Gentile F., Jneid H., Krieger E.V., Mack M., McLeod C. (2021). 2020 ACC/AHA Guideline for the Management of Patients with Valvular Heart Disease: Executive Summary: A Report of the American College of Cardiology/American Heart Association Joint Committee on Clinical Practice Guidelines. Circulation.

[10] Zhou S., Egorova N., Moskowitz G., Giustino G., Ailawadi G., Acker M.A., Gillinov M., Moskowitz A., Gelijns A. (2021). Trends in MitraClip, mitral valve repair, and mitral valve replacement from 2000 to 2016. J. Thorac. Cardiovasc. Surg..

[11] Pacini D., Murana G. (2021). Commentary: Trends in mitral valve interventions: The good, the bad, and the ugly. J. Thorac. Cardiovasc. Surg..

[12] Bonow R.O., O’Gara P.T., Adams D.H., Badhwar V., Bavaria J.E., Elmariah S., Hung J.W., Lindenfeld J., Morris A., Satpathy R. (2020). Multisociety expert consensus systems of care document 2019 AATS/ACC/SCAI/STS expert consensus systems of care document: Operator and institutional recommendations and requirements for transcatheter mitral valve intervention: A Joint Report of the American Association for Thoracic Surgery, the American College of Cardiology, the Society for Cardiovascular Angiography and Interventions, and The Society of Thoracic Surgeons. Catheter. Cardiovasc. Interv..

[13] Feldman T., Foster E., Glower D.D., Kar S., Rinaldi M.J., Fail P.S., Smalling R.W., Siegel R., Rose G.A., Engeron E. (2011). Percutaneous repair or surgery for mitral regurgitation. N. Engl. J. Med..

[14] Mauri L., Foster E., Glower D.D., Apruzzese P., Massaro J.M., Herrmann H.C., Hermiller J., Gray W., Wang A., Pedersen W.R. (2013). 4-Year Results of a Randomized Controlled Trial of Percutaneous Repair Versus Surgery for Mitral Regurgitation. J. Am. Coll. Cardiol..

[15] Sorajja P., Vemulapalli S., Feldman T., Mack M., Holmes D.R., Stebbins A., Kar S., Thourani V., Ailawadi G. (2017). Outcomes with Transcatheter Mitral Valve Repair in the United States: An STS/ACC TVT Registry Report. J. Am. Coll. Cardiol..

[16] Kalbacher D., Schäfer U., Bardeleben R.S.V., Eggebrecht H., Sievert H., Nickenig G., Butter C., May A.E., Bekeredjian R., Ouarrak T. (2019). Long-term outcome, survival and predictors of mortality after MitraClip therapy: Results from the German Transcatheter Mitral Valve Interventions (TRAMI) registry. Int. J. Cardiol..

[17] Melica B., Braga P., Ribeiro J., Pires-Morais G., Boa A.F., Guerreiro C., Caeiro D., Pereira R., Meerkin D., Fontes-Carvalho R. (2022). Transseptal Mitral Annuloplasty with the AMEND System. JACC Cardiovasc. Interv..

[18] Stone G.W., Lindenfeld J., Abraham W.T., Kar S., Lim D.S., Mishell J.M., Whisenant B., Grayburn P.A., Rinaldi M., Kapadia S.R. (2018). Transcatheter Mitral-Valve Repair in Patients with Heart Failure. N. Engl. J. Med..

[19] Obadia J.-F., Messika-Zeitoun D., Leurent G., Iung B., Bonnet G., Piriou N., Lefèvre T., Piot C., Rouleau F., Carrié D. (2018). Percutaneous Repair or Medical Treatment for Secondary Mitral Regurgitation. N. Engl. J. Med..

[20] Praz F., Spargias K., Chrissoheris M., Büllesfeld L., Nickenig G., Deuschl F., Schueler R., Fam N.P., Moss R., Makar M. (2017). Compassionate use of the PASCAL transcatheter mitral valve repair system for patients with severe mitral regurgitation: A multicentre, prospective, observational, first-in-man study. Lancet.

[21] Webb J.G., Hensey M., Szerlip M., Shafer U., Cohen G.N., Kar S., Makkar R., Kipperman R.M., Spargias K., O’Neill W.W. (2020). 1-Year Outcomes for Transcatheter Repair in Patients With Mitral Regurgitation From the CLASP Study. JACC Cardiovasc. Interv..

[22] Hausleiter J., Lim D.S., Gillam L.D., Zahr F., Chadderdon S., Rassi A.N., Makkar R., Goldman S., Rudolph V., Hermiller J. (2023). Transcatheter Edge-to-Edge Repair in Patients with Anatomically Complex Degenerative Mitral Regurgitation. J. Am. Coll. Cardiol..

[23] Geis N.A., Schlegel P., Heckmann M.B., Katus H.A., Frey N., Crespo López P., Raake P.W. (2022). One-year results following PASCAL-based or MitraClip-based mitral valve transcatheter edge-to-edge repair. ESC Heart Fail..

[24] Schneider L., Markovic S., Mueller K., Felbel D., Gerçek M., Friedrichs K., Stolz L., Rudolph V., Hausleiter J., Rottbauer W. (2022). Mitral Valve Transcatheter Edge-to-Edge Repair Using MitraClip or PASCAL: A Multicenter Propensity Score-Matched Comparison. JACC Cardiovasc. Interv..

[25] Lim D.S., Smith R.L., Gillam L.D., Zahr F., Chadderdon S., Makkar R., von Bardeleben R.S., Kipperman R.M., Rassi A.N., Szerlip M. (2022). Randomized Comparison of Transcatheter Edge-to-Edge Repair for Degenerative Mitral Regurgitation in Prohibitive Surgical Risk Patients. JACC Cardiovasc. Interv..

[26] El Sabbagh A., Reddy Y.N.V., Nishimura R.A. (2018). Mitral Valve Regurgitation in the Contemporary Era: Insights Into Diagnosis, Management, and Future Directions. JACC Cardiovasc. Imaging.

[27] Lesevic H., Karl M., Braun D., Barthel P., Orban M., Pache J., Hadamitzky M., Mehilli J., Stecher L., Massberg S. (2017). Long-Term Outcomes after MitraClip Implantation According to the Presence or Absence of EVEREST Inclusion Criteria. Am. J. Cardiol..

[28] Sherif M.A., Paranskaya L., Yuecel S., Kische S., Thiele O., D’Ancona G., Neuhausen-Abramkina A., Ortak J., Ince H., Öner A. (2017). MitraClip step by step; how to simplify the procedure. Neth. Heart J. Mon. J. Neth. Soc. Cardiol. Neth. Heart Found..

[29] Rogers J.H., Bolling S.F. (2021). Transseptal direct complete annuloplasty: Early experience. Ann. Cardiothorac. Surg..

[30] Messika-Zeitoun D., Nickenig G., Latib A., Kuck K.-H., Baldus S., Schueler R., La Canna G., Agricola E., Kreidel F., Huntgeburth M. (2019). Transcatheter mitral valve repair for functional mitral regurgitation using the Cardioband system: 1 year outcomes. Eur. Heart J..

[31] Rogers J.H., Boyd W.D., Smith T.W.R., Ebner A.A., Grube E., Bolling S.F. (2018). Transcatheter Annuloplasty for Mitral Regurgitation with an Adjustable Semi-Rigid Complete Ring: Initial Experience with the Millipede IRIS Device. Struct. Heart.

[32] Khatib D., Neuburger P.J., Pan S., Rong L.Q. (2022). Transcatheter Mitral Valve Interventions for Mitral Regurgitation: A Review of Mitral Annuloplasty, Valve Replacement, and Chordal Repair Devices. J. Cardiothorac. Vasc. Anesthesia.

[33] Siminiak T., Wu J.C., Haude M., Hoppe U.C., Sadowski J., Lipiecki J., Fajadet J., Shah A.M., Feldman T., Kaye D.M. (2012). Treatment of functional mitral regurgitation by percutaneous annuloplasty: Results of the TITAN Trial. Eur. J. Heart Fail..

[34] Lipiecki J., Siminiak T., Sievert H., Müller-Ehmsen J., Degen H., Wu J.C., Schandrin C., Kalmucki P., Hofmann I., Reuter D. (2016). Coronary sinus-based percutaneous annuloplasty as treatment for functional mitral regurgitation: The TITAN II trial. Open Heart.

[35] Witte K.K., Lipiecki J., Siminiak T., Meredith I.T., Malkin C.J., Goldberg S.L., Stark M.A., von Bardeleben R.S., Cremer P.C., Jaber W.A. (2019). The REDUCE FMR Trial: A Randomized Sham-Controlled Study of Percutaneous Mitral Annuloplasty in Functional Mitral Regurgitation. JACC Heart Fail..

[36] Guerrero M., Pursnani A., Narang A., Salinger M., Wang D.D., Eleid M., Kodali S.K., George I., Satler L., Waksman R. (2021). Prospective Evaluation of Transseptal TMVR for Failed Surgical Bioprostheses: MITRAL Trial Valve-in-Valve Arm 1-Year Outcomes. JACC Cardiovasc. Interv..

[37] Whisenant B., Kapadia S.R., Eleid M.F., Kodali S.K., McCabe J.M., Krishnaswamy A., Morse M., Smalling R.W., Reisman M., Mack M. (2020). One-Year Outcomes of Mitral Valve-in-Valve Using the SAPIEN 3 Transcatheter Heart Valve. JAMA Cardiol..

[38] Resor C.D. (2021). Transcatheter mitral valve interventions. Prog. Cardiovasc. Dis..

[39] Edwards L. (2022). PARTNER 3 Trial—Mitral Valve in Valve. https://www.clinicaltrials.gov/study/NCT03193801.

[40] Kalińczuk Ł., Mintz G.S., Chmielak Z., Rudziński P.N., Witkowski A. (2021). Intraprocedural assessment of valve geometry during transcatheter mitral valve replacement by large field-of-view intravascular ultrasound: A case report. Eur. Heart J. Case Rep..

[41] Kalinczuk L., Skotarczak W., Chmielak Z., Dabrowski M., Wolny R., Stoklosa P., Wozniak O., Hoffman P., Michalowska I., Demkow M. (2022). Abstract 12383: Large Field-of-View Intravascular Ultrasound for Peri-Procedural Assessment of Stent Frame Expansion after Transcatheter Heart Valve-in-Valve. Circulation.

[42] Guerrero M., Wang D.D., Pursnani A., Salinger M., Russell H.M., Eleid M., Chakravarty T., Ng M.H., Kodali S.K., Meduri C.U. (2021). Prospective Evaluation of TMVR for Failed Surgical Annuloplasty Rings: MITRAL Trial Valve-in-Ring Arm 1-Year Outcomes. JACC Cardiovasc. Interv..

[43] Xu B., Kocyigit D., Wang T.K.M., Tan C.D., Rodriguez E.R., Pettersson G.B., Unai S., Griffin B.P. (2022). Mitral annular calcification and valvular dysfunction: Multimodality imaging evaluation, grading, and management. Eur. Heart J. Cardiovasc. Imaging.

[44] Xu B., Saijo Y., Reyaldeen R.M., Brizneda M.V., Chan N., Gillinov A.M., Pettersson G.B., Unai S., Flamm S.D., Schoenhagen P. (2023). Novel Multi-Parametric Mitral Annular Calcification Score Predicts Outcomes in Mitral Valve Dysfunction. Curr. Probl. Cardiol..

[45] Fox C.S., Vasan R.S., Parise H., Levy D., O’Donnell C.J., D’Agostino R.B., Benjamin E.J. (2003). Mitral annular calcification predicts cardiovascular morbidity and mortality: The Framingham Heart Study. Circulation.

[46] Kato N., Guerrero M., Padang R., Amadio J.M., Eleid M.F., Scott C.G., Lee A.T., Pislaru S.V., Nkomo V.T., Pellikka P.A. (2022). Prevalence and Natural History of Mitral Annulus Calcification and Related Valve Dysfunction. Mayo Clin. Proc..

[47] Kato N., Pellikka P.A., Scott C.G., Lee A.T., Jain V., Eleid M.F., Alkhouli M.A., Reeder G.S., Michelena H.I., Pislaru S.V. (2022). Impact of mitral intervention on outcomes of patients with mitral valve dysfunction and annulus calcification. Catheter. Cardiovasc. Interv. Off. J. Soc. Card. Angiogr. Interv..

[48] Saijo Y., Chan N., Brizneda M.V., Lak H.M., Reyaldeen R.M., Gillinov A.M., Pettersson G.B., Unai S., Jellis C., Grimm R.A. (2021). Impact of Frailty and Mitral Valve Surgery on Outcomes of Severe Mitral Stenosis Due to Mitral Annular Calcification. Am. J. Cardiol..

[49] Guerrero M., Urena M., Himbert D., Wang D.D., Eleid M., Kodali S., George I., Chakravarty T., Mathur M., Holzhey D. (2018). 1-Year Outcomes of Transcatheter Mitral Valve Replacement in Patients with Severe Mitral Annular Calcification. J. Am. Coll. Cardiol..

[50] Yoon S.-H., Whisenant B.K., Bleiziffer S., Delgado V., Dhoble A., Schofer N., Eschenbach L., Bansal E., Murdoch D.J., Ancona M. (2018). Outcomes of transcatheter mitral valve replacement for degenerated bioprostheses, failed annuloplasty rings, and mitral annular calcification. Eur. Heart J..

[51] Guerrero M., Wang D.D., Himbert D., Urena M., Pursnani A., Kaddissi G., Iyer V., Salinger M., Chakravarty T., Greenbaum A. (2017). Short-term results of alcohol septal ablation as a bail-out strategy to treat severe left ventricular outflow tract obstruction after transcatheter mitral valve replacement in patients with severe mitral annular calcification. Catheter. Cardiovasc. Interv..

[52] Wang D.D., Guerrero M., Eng M.H., Eleid M.F., Meduri C.U., Rajagopal V., Yadav P.K., Fifer M.A., Palacios I.F., Rihal C.S. (2019). Alcohol Septal Ablation to Prevent Left Ventricular Outflow Tract Obstruction during Transcatheter Mitral Valve Replacement: First-in-Man Study. JACC Cardiovasc. Interv..

[53] Khan J.M., Babaliaros V.C., Greenbaum A.B., Foerst J.R., Yazdani S., McCabe J.M., Paone G., Eng M.H., Leshnower B.G., Gleason P.T. (2019). Anterior Leaflet Laceration to Prevent Ventricular Outflow Tract Obstruction during Transcatheter Mitral Valve Replacement. J. Am. Coll. Cardiol..

[54] Guerrero M., Wang D.D., Eleid M.F., Pursnani A., Salinger M., Russell H.M., Kodali S.K., George I., Bapat V.N., Dangas G.D. (2021). Prospective Study of TMVR Using Balloon-Expandable Aortic Transcatheter Valves in MAC: MITRAL Trial 1-Year Outcomes. JACC Cardiovasc. Interv..

[55] Makkar R.R., Fontana G.P., Jilaihawi H., Kapadia S., Pichard A.D., Douglas P.S., Thourani V.H., Babaliaros V.C., Webb J.G., Herrmann H.C. (2012). Faculty Opinions recommendation of Transcatheter aortic-valve replacement for inoperable severe aortic stenosis. N. Engl. J. Med..

[56] Guerrero M., Vemulapalli S., Xiang Q., Wang D.D., Eleid M., Cabalka A.K., Sandhu G., Salinger M., Russell H., Greenbaum A. (2020). Thirty-Day Outcomes of Transcatheter Mitral Valve Replacement for Degenerated Mitral Bioprostheses (Valve-in-Valve), Failed Surgical Rings (Valve-in-Ring), and Native Valve With Severe Mitral Annular Calcification (Valve-in-Mitral Annular Calcification) in the United States: Data From the Society of Thoracic Surgeons/American College of Cardiology/Transcatheter Valve Therapy Registry. Circ. Cardiovasc. Interv..

[57] Urena M., Brochet E., LeComte M., Kerneis C., Carrasco J.L., Ghodbane W., Abtan J., Alkhoder S., Raffoul R., Iung B. (2018). Clinical and haemodynamic outcomes of balloon-expandable transcatheter mitral valve implantation: A 7-year experience. Eur. Heart J..

[58] Sorajja P., Moat N., Badhwar V., Walters D., Paone G., Bethea B., Bae R., Dahle G., Mumtaz M., Grayburn P. (2019). Faculty Opinions recommendation of Initial feasibility study of a new transcatheter mitral prosthesis: The first 100 patients. J. Am. Coll. Cardiol..

[59] Dahle G. (2020). Current Devices in TMVI and Their Limitations: Focus on Tendyne. Front. Cardiovasc. Med..

[60] Muller D.W., Sorajja P., Duncan A., Bethea B., Dahle G., Grayburn P., Babaliaros V., Guerrero M., Thourani V.H., Bedogni F. (2021). 2-Year Outcomes of Transcatheter Mitral Valve Replacement in Patients With Severe Symptomatic Mitral Regurgitation. J. Am. Coll. Cardiol..

[61] Sorajja P., Gössl M., Babaliaros V., Rizik D., Conradi L., Bae R., Burke R.F., Schäfer U., Lisko J.C., Riley R.D. (2019). Novel Transcatheter Mitral Valve Prosthesis for Patients with Severe Mitral Annular Calcification. J. Am. Coll. Cardiol..

[62] Gössl M., Thourani V., Babaliaros V., Conradi L., Chehab B., Dumonteil N., Badhwar V., Rizik D., Sun B., Bae R. (2022). Early outcomes of transcatheter mitral valve replacement with the Tendyne system in severe mitral annular calcification. Eurointervention J. EuroPCR Collab. Work. Group Interv. Cardiol. Eur. Soc. Cardiol..

[63] Medical D.A. (2023). Clinical Trial to Evaluate the Safety and Effectiveness of Using the Tendyne Transcatheter Mitral Valve System for the Treatment of Symptomatic Mitral Regurgitation. https://classic.clinicaltrials.gov/ct2/show/NCT03433274.

[64] Bernard S., Yucel E. (2019). Paravalvular Leaks-From Diagnosis to Management. Curr. Treat. Options Cardiovasc. Med..

[65] Cruz-Gonzalez I., Rama-Merchan J.C., Rodríguez-Collado J., Martín-Moreiras J., Diego-Nieto A., Barreiro-Pérez M., Sánchez P.L. (2017). Transcatheter closure of paravalvular leaks: State of the art. Neth. Heart J. Mon. J. Neth. Soc. Cardiol. Neth. Heart Found..

[66] Dávila-Román V.G., Waggoner A.D., Kennard E.D., Holubkov R., Jamieson W., Englberger L., Carrel T.P., Schaff H.V. (2004). Prevalence and severity of paravalvular regurgitation in the Artificial Valve Endocarditis Reduction Trial (AVERT) echocardiography study. J. Am. Coll. Cardiol..

[67] Hammermeister K., Sethi G.K., Henderson W.G., Grover F.L., Oprian C., Rahimtoola S.H. (2000). Outcomes 15 years after valve replacement with a mechanical versus a bioprosthetic valve: Final report of the Veterans Affairs randomized trial. J. Am. Coll. Cardiol..

[68] Ionescu A., Fraser A.G., Butchart E.G. (2003). Prevalence and clinical significance of incidental paraprosthetic valvar regurgitation: A prospective study using transoesophageal echocardiography. Heart.

[69] Genoni M., Franzen D., Vogt P., Seifert B., Jenni R., Künzli A., Niederhäuser U., Turina M. (2000). Paravalvular leakage after mitral valve replacement: Improved long-term survival with aggressive surgery?. Eur. J. Cardio-Thorac. Surg..

[70] Gafoor S., Franke J., Bertog S., Lam S., Vaskelyte L., Hofmann I., Sievert H., Matic P. (2015). A Quick Guide to Paravalvular Leak Closure. Interv. Cardiol..

[71] Gafoor S., Steinberg D.H., Franke J., Bertog S., Vaskelyte L., Hofmann I., Sievert H. (2014). Tools and Techniques—Clinical: Paravalvular leak closure. Eurointervention J. EuroPCR Collab. Work. Group Interv. Cardiol. Eur. Soc. Cardiol..

[72] García E., Sandoval J., Unzue L., Hernandez-Antolin R., Almeria C., Macaya C. (2012). Paravalvular leaks: Mechanisms, diagnosis and management. Eurointervention J. EuroPCR Collab. Work. Group Interv. Cardiol. Eur. Soc. Cardiol..

[73] Sorajja P., Cabalka A.K., Hagler D.J., Rihal C.S. (2011). Percutaneous repair of paravalvular prosthetic regurgitation: Acute and 30-day outcomes in 115 patients. Circ. Cardiovasc. Interv..

[74] Ruiz C.E., Jelnin V., Kronzon I., Dudiy Y., Del Valle-Fernandez R., Einhorn B.N., Chiam P.T., Martinez C., Eiros R., Roubin G. (2011). Clinical Outcomes in Patients Undergoing Percutaneous Closure of Periprosthetic Paravalvular Leaks. J. Am. Coll. Cardiol..

[75] Krishnaswamy A., Tuzcu E.M., Kapadia S.R. (2011). Three-dimensional computed tomography in the cardiac catheterization laboratory. Catheter. Cardiovasc. Interv. Off. J. Soc. Card. Angiogr. Interv..

[76] Kumar R., Jelnin V., Kliger C., Ruiz C.E. (2013). Percutaneous Paravalvular Leak Closure. Cardiol. Clin..

[77] Cruz-Gonzalez I., Antunez-Muiños P., Lopez-Tejero S., Sanchez P.L. (2022). Mitral Paravalvular Leak: Clinical Implications, Diagnosis and Management. J. Clin. Med..

[78] García E., Arzamendi D., Jimenez-Quevedo P., Sarnago F., Martí G., Sanchez-Recalde A., Lasa-Larraya G., Sancho M., Iñiguez A., Goicolea J. (2017). Outcomes and predictors of success and complications for paravalvular leak closure: An analysis of the SpanisH real-wOrld paravalvular LEaks closure (HOLE) registry. Eurointervention J. EuroPCR Collab. Work. Group Interv. Cardiol. Eur. Soc. Cardiol..

[79] Calvert P.A., Northridge D.B., Malik I.S., Shapiro L., Ludman P., Qureshi S.A., Mullen M., Henderson R., Turner M., Been M. (2016). Percutaneous Device Closure of Paravalvular Leak: Combined Experience from the United Kingdom and Ireland. Circulation.

[80] Onorato E.M., Muratori M., Smolka G., Zakarkaite D., Mussayev A., Christos C.P., Bauer F., Gandet T., Luca G., Martinelli M.D. (2020). Midterm procedural and clinical outcomes of percutaneous paravalvular leak closure with the Occlutech Paravalvular Leak Device. Eurointervention J. EuroPCR Collab. Work. Group Interv. Cardiol. Eur. Soc. Cardiol..

